# Effect of etch-and-rinse and self-etch modes in universal adhesives on push-out bond strength of fiber post

**DOI:** 10.4317/jced.59564

**Published:** 2022-08-01

**Authors:** Citra Kusumasari, Anggraini Margono, Dimas-Raihan Aditya, Ahmed Abdou

**Affiliations:** 1 Assistant Professor, Department of Conservative Dentistry, Faculty of Dentistry, Universitas Indonesia, Jakarta, Indonesia; 2 Associate Professor, Department of Conservative Dentistry, Faculty of Dentistry, Universitas Indonesia, Jakarta, Indonesia; 3 Undergraduate Program, Faculty of Dentistry, Universitas Indonesia, Jakarta, Indonesia; 4 Assistant Professor, Prosthetic Dentistry Department, Faculty of Dentistry, King Salman International University, South Sinai, Egypt

## Abstract

**Background:**

Nowadays, the universal adhesive become more popular among clinicians due to its simple procedure. The application of universal adhesive on root canal dentin prior the self-adhesive resin cement may increase bond-strength between fiber post and dentin. The objective of this study was to evaluate the effect of different etching modes (etch-and-rinse and self-etch) to universal adhesives on push-out bond strength of fiber post.

**Material and Methods:**

Thirty extracted sound human lower premolars were randomly divided into three groups based on adhesives systems prior to fiber post cementation; two-step etch-and-rinse (group A, Adper Scotchbond), etch-and-rinse universal (group B, Prime & Bond Universal), and self-etch universal (group C, Prime & Bond Universal). After adhesive application, self-adhesive resin cement was filled to the prepared root canal (Smart CEM2, Dentsply) for fiber post cementation. The adhesion between the fiber post and root canal walls was investigated using push-out test after 24 h water storage at 37◦C and the modes of failure were determined under SEM. Data were analyzed using two-way ANOVA test and the Bonferroni test was used to compare values among the three adhesives groups, followed by Tukey HSD for multiple comparisons. Furthermore, Weibull parameters were calculated for tested groups.

**Results:**

Universal adhesive with self-etch mode significantly improved bond-strength compared to the two-step etch-and-rinse group (*p*<0.05). The coronal part has higher bond strength than the apical section (*p*<0.05). However, the bond-strength in two-step etch-and-rinse and etch-and-rinse universal was not significantly different. Self-etch mode showed higher bond strength compared to etch-and-rinse universal adhesive in the apical root section (*p*<0.05). SEM revealed that all tested groups predominantly had an adhesive failure (*p*>0.05).

**Conclusions:**

Self-etch mode in universal adhesive system were effectively improved the push-out bond strength of fiber post to root canal dentin, especially in apical root section.

** Key words:**Push-out bond-strength, self-adhesive resin cement, self-etch adhesive, total-etch.

## Introduction

Successful endodontic treatment is based on an ideal access cavity preparation, biomechanical preparation, and obturation. However, the survival of the endodontically treated teeth is not only affected by the endodontic treatment quality but depends on creating an optimal seal between internal and external root restorations (1). Irrigation and disinfection products, as well as the adhesive system and coronal restoration, can all have an impact on dentin bonding (2). Additionally, fiber post is an essential clinical option in dentistry and an adequate material in restoring endodontically treated teeth. Glass-fiber posts offer two significant advantages, first is the elastic modulus being similar to that of dentin (3), and the second is its simple procedure by cementing it using an adhesive technique (4). There are two contemporary resin cements based on the adhesive approach, regular resin cements and self-adhesive or self-etching resin cements. The latter cements is self-adherent and no acid or primers application on the dentin substrate prior to the adhesive/bonding procedure (5). Moreover, a number of resin cements have been introduced as one-component “universal adhesive cements”, it can bond to an untreated tooth surface that has not been micro-abraded or pretreated with an etchant, primer, or bonding agent (6).

To achieve a self-adhesive reaction of the self-adhesive resin cement to the tooth structure, a methacrylate monomers with phosphoric acid groups were added. It will effect on a low pH value and hydrophilic properties in the beginning of the setting. Furthermore, the negatively charged groups of the monomer bind to Ca2+ ions of the tooth and in combination with the alkaline part of the fillers then result in a neutralization reaction (7). However, not all self-adhesive resin cements can be adequate alternative to regular resin cements, due to lower bond strength mean compared to regular resin cements (8).

Application of universal adhesive on root canal dentin prior the self-adhesive resin cement may increase bond-strength between fiber post and dentin. Nowadays, the universal adhesive become more popular among the clinicians, due to the increasing of simpler and more versatile adhesives, moreover, this adhesive can be used in etch-and-rinse or self-etch modes (9). The etch-and-rinse modes in universal adhesive may overcome the mildness pH of one-step self-etch adhesive system by using the etching procedure prior the adhesive. The other modes; self-etch mode can bond chemically to hydroxyapatite in dentin due to its methacryloyloxydecyl dihydrogen phosphate (10-MDP) monomer and forming hydrolytically sTable calcium on hydroxyapatite in the form of nanolayering (10).

The objective of this study was therefore to evaluate the effect of different etching modes (etch-and-rinse and self-etch) in universal adhesives on push-out bond strength of fiber post cementation using self-adhesive resin cement, and to compare them with two-step etch-and-rinse adhesive. Push-out bond strength test is a method to evaluate the bond strength in root canals with a high c-factor and high-stress generation directed toward the bonding area (11). Additionally, scanning electron microscopy (SEM) was used to evaluate the precise failure mode on the fractured specimen surfaces. The null hypothesis was that there would be no difference in the bond strength between the fiber post and root canal walls in coronal and apical part of different etching modes (etch-and-rinse and self-etch) in universal adhesives and two-step etch-and-rinse adhesive for fiber post cementation.

## Material and Methods

-Specimen preparation for bond strength evaluation

For bond strength evaluation using the push-out test, thirty sound extracted human lower first premolars with one root canal were used (Number 52/ Ethical Approval/ FKGUI/ VIII/ 2021, protocol number: 010580821). Specimens with less than 20 mm total length, root resorption, and has root curvature were discarded. Then, the teeth were randomly divided into three adhesives systems groups prior to fiber post cementation; two-step etch-and-rinse (group A, control, Adper Scotchbond, 3M ESPE, St. Paul, MN, USA), etch-and-rinse universal (group B, Prime & Bond Universal, Denstply Sirona, Charlotte, NC, USA), and self-etch universal (group C, Prime & Bond Universal, Denstply Sirona, Charlotte, NC, USA).

The specimens were decoronated at the cervico-enamel junction then the root canal treatment and obturation were done using crown down preparation with hand use tapered file up to size F3 (30,0.09) (ProTaper, Denstply Sirona, Charlotte, NC, USA) and single cone obturation. After 24 hours stored in water at 37◦C, 8 mm fiber post space preparation was done using post drill (Ø150 mm, FibreKleer, Pentron clinical, CA). The adhesive systems application for all groups were according to the manufacturers’ instructions. Soon after, the application of self-adhesive resin cement into the root canal, the fiber post was inserted (Ø150 mm, smooth parallel, FibreKleer post, Pentron, clinical, CA) and light cured for 30 s (EliparTM S10 LED Curing Light, 3M ESPE, St. Paul, MN, USA) from the coronal tip of fiber post. The root canal then cut into 2 coronal discs and 2 apical discs in 1.5 mm thickness. The composition of adhesive system and self-adhesive resin cement are presented in Table 1.

**Table 1:**
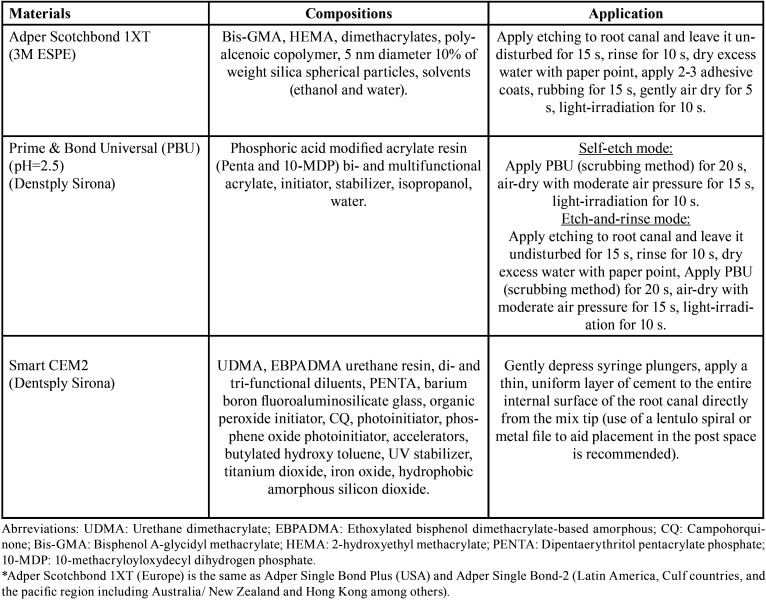
The composition of adhesive system and self-adhesive resin cement used and application procedure.

-Push-out test using universal testing machine

The specimens were tested using push-out test by sectioned the coronal and apical thirds perpendicularly to the root axis using a low-speed diamond wafering blade under water irrigation. Therefore, 4 slices of approximately 1.5 mm for each tooth and 120 specimens in total were obtained. After cutting procedure, the specimens were placed on a metal base made of stainless steel, having a central hole of 2 mm in diameter. Then, the loading was applied on the post, on the root slice apical face using a tip with 1.0 mm in diameter and coupled to a universal testing machine (Shimadzu, AG-5000E) at 1 mm/min, until the post was displaced. The bond strength to the post displacement was obtained automatically by the formula using trapezium lite x software which is resulted in MPa. The remaining disc specimens were then observed using scanning electron microscopy.

-Morphological observation of failure mode using scanning electron microscopy

**Figure 1:**
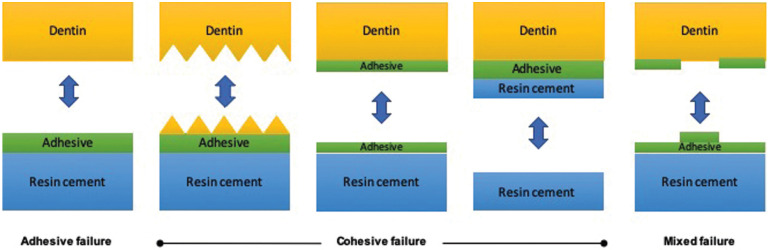
The failure modes categories.

After the specimens were sputter-coated with carbon, each specimen was evaluated using a scanning electron microscopy (JSM-5310, JEOL, Tokyo, Japan) at 500x magnification under the voltage of 20.0 kV to determine the failure modes. The following failure modes were distinguished based on (Fig. 1): (a) adhesive failure (80-100% failure occurred between adhesive resin and dentin), (b) cohesive failure (80-100% of the failure occurred in the underlying dentin, in the adhesive resin and/or overlying cement), (c) mixed failure (adhesive failure and cohesive failure in the adhesive resin and/or dentin).

-Statistical analysis

The Kolmogorov-Smirnov test which was used to check the distribution normality showed parametric distribution of the bond strength data. Therefore, data were analyzed using two-way ANOVA test to compare values among the three different adhesives groups and two root section. Multiple comparisons were done using Tukey HSD (α=0.05). For failure mode analysis, Kruskal Wallis test used to compare between adhesives and two root section. All calculations were performed using the IBM SPSS software (Version 26, IBM Corp., Armonk, NY, USA). Furthermore, Weibull parameters were calculated using maximum likelihood estimation, and 95% confidence intervals were calculated with Monte Carlo simulations. The different groups were compared at the characteristic strength (63.2% probability of failure) (R4.1, R foundation for statistics, Vienna).

## Results

-Push-out bond strength test

**Figure 2:**
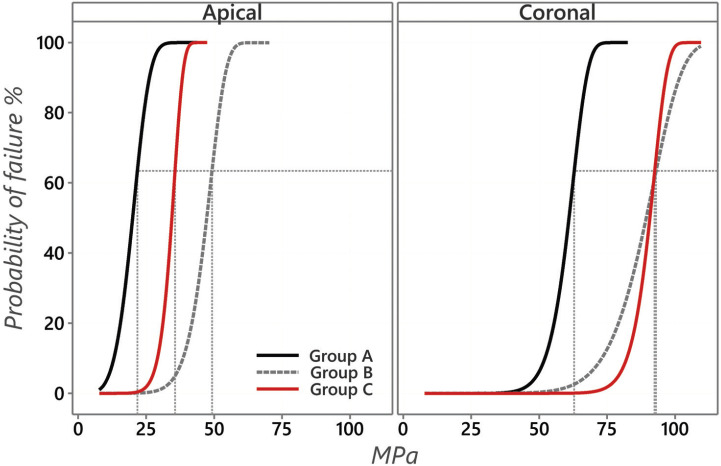
Weibull Survival plot with a horizontal reference line at 63.2% failure probability and vertical reference line intersecting with survival curves for coronal and apical section.

The push-out bond strength data are presented in Table 2 and Table 3. Two-way ANOVA showed that different adhesives and root section had a significant effect on push-out bond strength (both, *p*<0.001). While the interaction between both variables showed an insignificant effect at *p*=0.065. Moreover, the statistical analysis revealed higher significantly difference for bond strength in the coronal compared to apical root section (*p*<0.001). For both root section, A (control, two-step etch-and-rinse) showed the lowest significant bond-strength compared to group group B (etch-and-rinse universal) and group C (self-etch universal) (*p*<0.05). There was no significant difference in the bond-strength between group B and group C for the coronal (*p*=1.00) and apical root section (*p*=0.052). However, Weibull analysis results are presented in Table 4 and Figure 2. Group C (self-etch universal) showed a significant higher characteristics strength (*p*<0.05) compared to group B (etch-and-rinse universal) for apical root section; which was different than ANOVA finding.

**Table 2:**
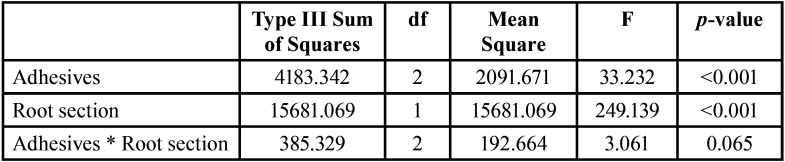
One-way ANOVA for push-out bond strength test.

**Table 3:**
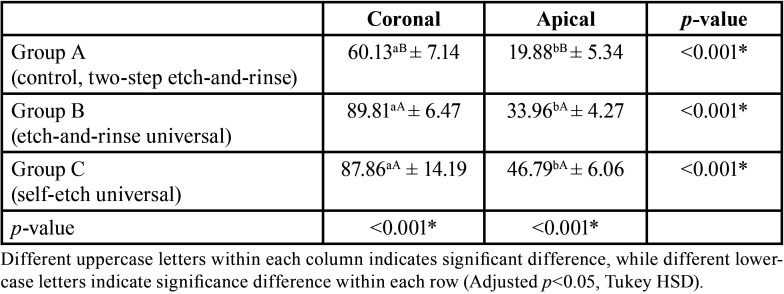
Multiple comparison between groups for push-out bond strength test.

**Table 4:**
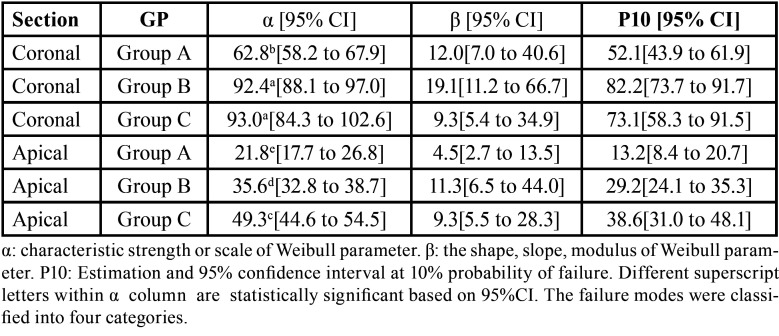
Weibull analysis for push-out bond strength test for different tested groups.

-Failure mode analysis

**Figure 3:**
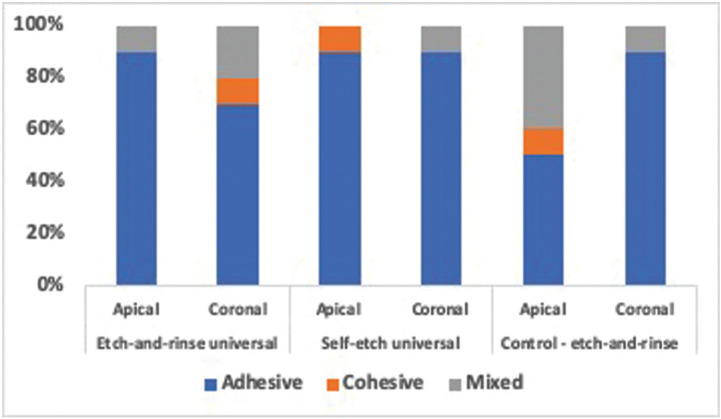
The failure modes result for all tested adhesives and root sections.

**Figure 4:**
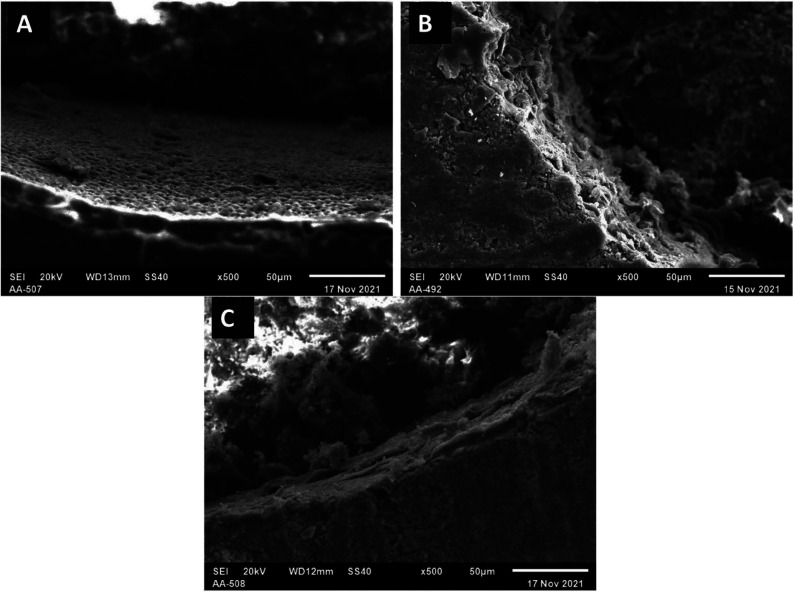
The SEM representative images of failure modes in 500x magnification (A: adhesive failure, B: cohesive failure, C: mixed failure).

The failure mode on the fractured specimen surfaces were observed at one magnification 500x and analysed by chi-square test. There were no clear differences in failure mode characteristics between the control group and all tested adhesive for both root sections (*p*=0.351) (Fig. 3). The SEM images of of failure modes are presented in Fig. 4.

## Discussion

The universal adhesives become popular due to their simplicity and more versatile adhesives. In this study, Prime and Bond Universal adhesive was used, which is contained specific monomers that bond ionically to calcium hydroxyapatite. The methacryloyloxydecyl-dihydrogen-phosphate (10-MDP) functional monomer has the potential to bond chemically to tooth tissues through the formation of non-soluble Ca2+ salts. Moreover, their acidic nature (esters of phosphoric acid) gives them the potential to etch and demineralize tooth tissues, which makes them possible for use in adhesives that require etch-and-rinse, self-etch, and selective-etch mode (12).

In all tested groups, the coronal root section demonstrated significantly higher bond strength than the apical section (*p*<0.001). It may be attributed to sufficient solvent evaporation from the all tested adhesives in the post cavity and resulting in no formation of voids in the adhesive layer in the coronal section (13). The universal adhesives for both modes revealed significantly higher bond strength than the control group (two-step etch-and-rinse adhesives) (*p*<0.05). There is a siginificant difference in the bond strength between universal adhesives etch-and-rinse mode and self-etch mode for the apical root section. Therefore, in this study, the null hypothesis was rejected.

In associated with the number of dentinal tubules being higher in the coronal root section than apical (14), the self-adhesive resin cement used in this study produced bond-strength values in the coronal root section that were higher than the apical root section. Additionally, the etching time and cleaning of the canal could prevent the adhesive to make penetration, producing low bond strength areas (15-17).

The adhesive systems used in the cementation of the fiber posts may contribute to the differences in bond strength. The control group (two-step etch-and-rinse adhesive) presented a lower bond strength than universal adhesives in both modes due to the acid etching removing both the smear layer and the organic content of dentin. It may damage the bond between cement and dentin, then reduce the bond strength (18). Following the SEM observation results in two-step etch-and-rinse adhesive group, predominantly had adhesive and mixed failure modes. The failure may be correlated with the incapability of the adhesive to infiltrate into the exposed collagen-fibril network deeper than the over-etched dentin, resulting in nanoleakge, and when associated with water sorption and hydrolysis; it will produce bond-degradation on dentin (19).

Similarly, with the previous study, the push-out bond strength of two modes (etch-and-rinse and self-etch) in universal adhesive was a significant difference (20). The self-etch mode in universal adhesive improved the bond strength in the apical section. The use of etching prior to the universal adhesive does not cause adequate exposure of the collagen fibers due to the incapability of the etching to flow entirely into the apical section of the root canal. Then, etching could not be wholly rinsed away, and residual etching may produce low-pH-related inhibition of polymerization of resin-based materials (21). In corroborating with SEM observation, the failure mode in the universal adhesive for both root sections was dominantly adhesive failures, probably related to the difficulty of the bonding agent to infiltrate into the exposed collagen dentin completely and decreased mechanical properties of the dentin (22).

When universal adhesives are applied in self-etch mode, they are basically one-step self-etch adhesives. Despite the poor clinical performance of traditional one-step self-etch adhesives due to their strong acidity, the improvement of self-etch adhesive with mild acidity shown improved performance. As the pH of most universal adhesives is more than 2.0 (mild acidity), the improved bonding rates in mild self-etch adhesives may happen as well in the universal adhesives (23). Therefore, significantly higher bond strength in universal adhesives in coronal and apical root sections compared to two-step etch-and-rinse adhesive is shown in this study. The other reason for the better bonding in self etch mode of universal adhesives is the solvent evaporation time upon applying of the adhesive on dentin root canal for 15 seconds. Thus, the residual water may not be left and there is no chance that hydrolytic degradation of polymers and collagen will happen (24). In addition, the scrubbing method when applying universal adhesives may enhance the penetration of monomer into the dentin and improve the bond strength.

The etch-and-rinse mode was used in this study as the other adhesion strategy for universal adhesive. The bond strength observation is higher than the two-step etch-and-rinse adhesive, but as same as self-etch mode in universal adhesive. In agreement with the previous study, the bond strength on dentin of universal adhesives was not affected by the adhesion strategy or dentin moisture (25). Additionally, the apical section part using etch-and-rinse mode universal adhesive has lower bond strength compared to self-etch mode. Even though it was not significant, the etching step may produced a lower value in the etch-and-rinse mode. The flowability of the etching gel in the root canal may fail in collagen fibers exposure. Furthermore, the etching gel cannot be thoroughly washed away and the residual may cause low-pH-related inhibition of polymerization of resin-based materials (26).

The universal adhesive in self-etch mode is preferable to use in the clinical situation due to the simplicity and tends to show higher bond strength for the coronal and apical section part while cementing fiber post using self-adhesive resin cement. The immediate result of bond strength was recognized as the limitation of this study. Further research is needed for long-term bonding observation with another dentin substrate using universal adhesives.

## Conclusions

Within the limitations of this study, we conclude that the universal adhesive in self-etch mode applied to root canal dentin prior fiber post cementation can improve bond strength to root canal dentin. Additionally, the scrubbing method when apply the adhesive and 15 seconds solvent evaporation may enhance bond strength in universal adhesive.
